# 3D Forest: An application for descriptions of three-dimensional forest structures using terrestrial LiDAR

**DOI:** 10.1371/journal.pone.0176871

**Published:** 2017-05-04

**Authors:** Jan Trochta, Martin Krůček, Tomáš Vrška, Kamil Král

**Affiliations:** 1 The Silva Tarouca Research Institute, Department of Forest Ecology, Lidicka, Brno, Czech Republic; 2 Mendel University in Brno, Faculty of Forestry and Wood Technology, Department of Geoinformation Technologies, Zemedelska, Brno, Czech Republic; University of the Chinese Academy of Sciences, CHINA

## Abstract

Terrestrial laser scanning is a powerful technology for capturing the three-dimensional structure of forests with a high level of detail and accuracy. Over the last decade, many algorithms have been developed to extract various tree parameters from terrestrial laser scanning data. Here we present 3D Forest, an open-source non-platform-specific software application with an easy-to-use graphical user interface with the compilation of algorithms focused on the forest environment and extraction of tree parameters. The current version (0.42) extracts important parameters of forest structure from the terrestrial laser scanning data, such as stem positions (*X*, *Y*, *Z*), tree heights, diameters at breast height (DBH), as well as more advanced parameters such as tree planar projections, stem profiles or detailed crown parameters including convex and concave crown surface and volume. Moreover, 3D Forest provides quantitative measures of between-crown interactions and their real arrangement in 3D space. 3D Forest also includes an original algorithm of automatic tree segmentation and crown segmentation. Comparison with field data measurements showed no significant difference in measuring DBH or tree height using 3D Forest, although for DBH only the Randomized Hough Transform algorithm proved to be sufficiently resistant to noise and provided results comparable to traditional field measurements.

## Introduction

Much forest ecosystem research is based on spatially oriented data. Research on forest dynamics commonly makes use of large census plots, where the position and size of every tree individual are measured and recorded [1]. These observations are fundamentally two-dimensional, trees being represented as points with *X*, *Y* coordinates of the tree base and other parameters (e.g. species, diameter in breast height—DBH, height) only recorded in a database. However, forests are intrinsically three-dimensional systems. Canopy disturbances, tree regeneration, tree growth and competition (especially aboveground competition for light) all take place in real 3D space. These processes cannot be explicitly represented in two-dimensional forest plots.

The technology of terrestrial laser scanning (TLS) undoubtedly has the potential to change this state of affairs and bring real 3D insights to research in forest ecology and dynamics. It has great promise for collecting spatial information in forests because of its excellent measurement precision, short acquisition time, and level of detail [2]. TLS is capable of acquiring levels of detail far beyond the capabilities of airborne laser scanning [3, 4], and thus may be used to describe forest stand vegetation at the level of individual trees including juvenile sub-canopy trees [5].

The output of TLS data preprocessing are registered and aligned point clouds with millions of points oriented in 3D space with millimeter accuracy. This specific data format requires specific methods of processing. Due to the extensive amounts of data and their high information potential, the automated processing of TLS point clouds is of crucial importance. Numerous algorithms have been introduced during past decade(s), with early studies focusing on basic tree parameters such as tree height, DBH and position [6] and recent works dealing with more advanced issues such as crown shape and dimensions [7], light propagation in forest gaps [8] and individual-specific estimates of woody biomass [9]. The recent development of several applications for extraction of various tree parameters from TLS point clouds (e.g. SimpleTree [10], CompuTree [11], LiForest [12] or AutoStem [13]) demonstrates that using TLS has great potential to help foresters and forest researchers in detailed tree analysis. Each of these applications has been suited for specific purposes and has their pros and cons. AutoStem is a commercial product focused on tree descriptions and forest inventory from the viewpoint of timber production. SimpleTree is an open source application that is optimal for detailed single-tree description and parametrization, providing more parameters for a single tree than other applications. CompuTree has its role as a platform for other algorithms associated with laser scanning. A variety of tree parameters or segmentation algorithms can be accessed through plugins. However, its graphical interface is designed for advanced user with detailed knowledge of the underlying algorithms, which may be limiting for many potential users. LiForest is more focused on plot level applications and was primarily designed for processing of airborne laser scanning (ALS) data; its use for satisfactory TLS data processing is as yet rather limited. Moreover, to the best of our knowledge, none of these software packages focuses on the parametrization of tree crowns and their spatial arrangement and interactions in the real 3D neighborhood, which are very important issues from the viewpoint of forest ecology and silviculture.

Therefore, we introduce 3D Forest, a software application for describing forest 3D structure through parametrization of individual trees and their crowns. The application is not platform-specific, and has an easy-to-use graphical interface (GUI) suitable for non-experts in TLS data processing. It provides a free, open-source solution for computing the following tree parameters: tree base position, DBH, tree height, stem curve, tree planar projection and crown parameters like: crown centroid, crown position deviation, crown base height, crown dimensions (height, length, width), crown volume and surface using convex hull or concave hull or volume and position of crown intersections. It is also capable of producing a detailed digital terrain model (DTM) of the study plot.

In the following sections, we introduce the algorithms employed for the extraction of terrain and individual tree parameters, briefly describe the workflow in 3D Forest, and present a comparison of two principal TLS derived tree parameters with conventional field measurements. We conclude by describing current and future developments to be incorporated in future versions of 3D Forest aimed at forest ecology and forest dynamics research in particular.

## 3D Forest workflow

**Fig 1:**
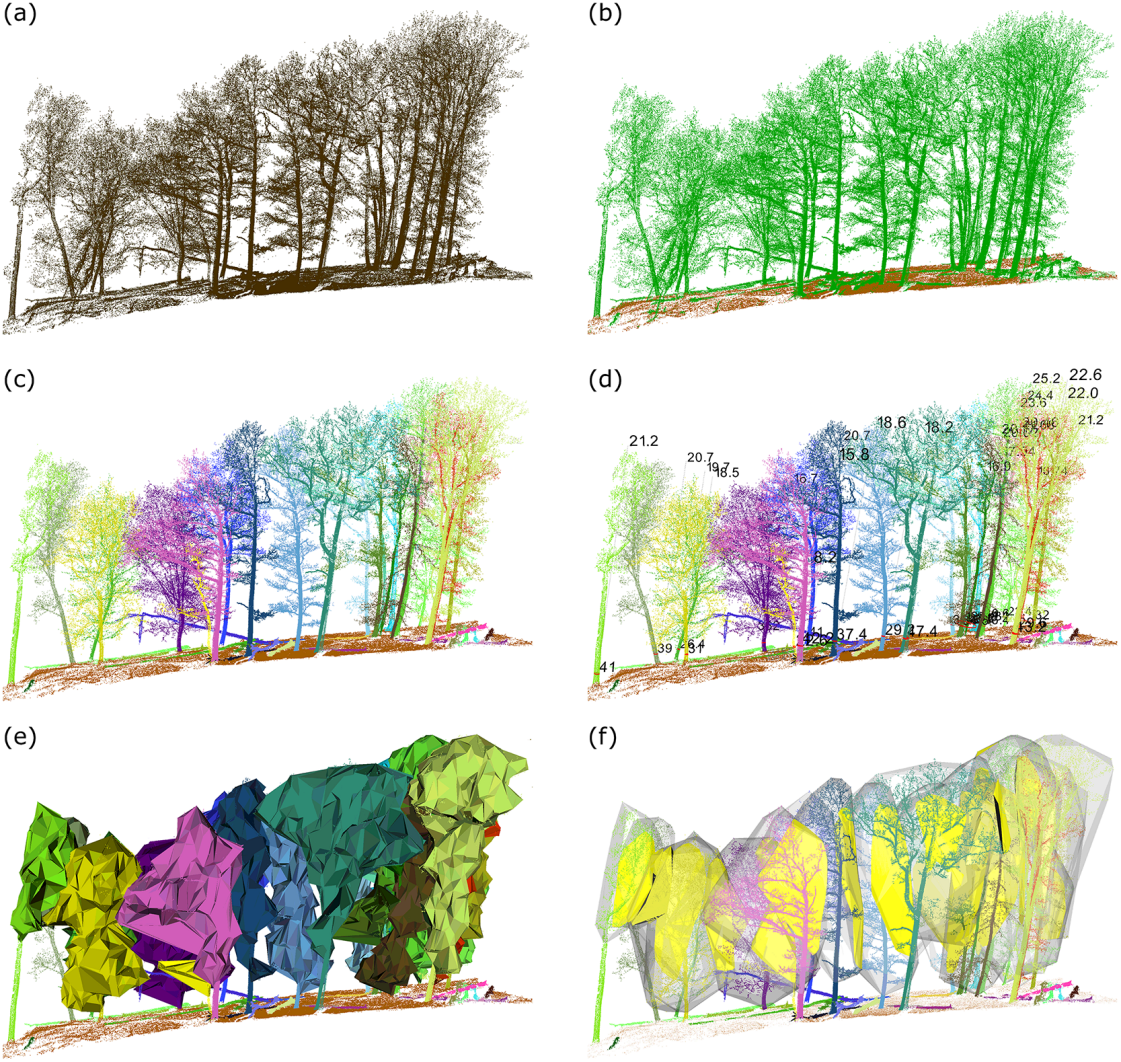
Demonstration of the 3D Forest workflow on a small sub-sample of the VPFDP (20m x 40m transect). (a) TLS data imported into 3D Forest (i.e. the Base cloud) prior to any segmentation; (b) automatically segmented Terrain cloud (brown) and Vegetation cloud (green) using the octree search method, refined by manual adjustment; (c) individual trees segmented into Tree clouds displayed in random colors; (d) DBH and tree height displayed for each tree; (e) concave hulls of tree crowns; (f) crowns represented by 3D convex hulls and their mutual intersections (in yellow).

To better demonstrate the workflow of 3D Forest and its outputs, we present an example of TLS data processing using a small subplot (20m x 40m) of a larger study site known as the Velká Pleš Forest Dynamics Plot (VPFDP) (Fig 1).

Prior to importing into 3D Forest, the data scanned from multiple scanner positions are fitted and registered in the proprietary software usually provided with the ground-based laser scanner. 3D Forest can import data in the following formats: txt, xyz, pcd, pts, ptx and las. In the 3D Forest workflow, the imported point cloud prior to any segmentation is called the Base cloud (Fig 1a).

The Base cloud is then separated into two parts: i) the points representing the terrain surface, i.e. the Terrain cloud; and ii) all other points, which in forests usually represent vegetation and therefore called the Vegetation cloud (Fig 1b).

The next step is segmentation of the Vegetation cloud into individual trees–i.e. Tree clouds (Fig 1c). This is done automatically by the algorithm described below (section 3.2.) with manual adjustment possible.

Individual Tree clouds are the subject of further automatic processing. Various tree and crown parameters can be extracted, e.g. DBH and tree height (Fig 1d), and/or crown surface, volume and other crown parameters can be estimated (Fig 1e). Between-crown interactions can be quantified by crown convex hull intersections (Fig 1f) and their parameters. All extracted tree parameters are simultaneously visualized in the 3D Forest viewer, which allows a direct visual check of their fit with appropriate tree clouds. Results can be exported as a table of extracted tree parameters, images of the viewer or segmented point clouds (e.g. terrain cloud or tree clouds) for further analyses in other software. The geometry of tree planar projections can be exported in a.txt file of polygon vertex coordinates and imported as a polygon vector layer into common GIS software.

## Algorithms used in 3D Forest

The application 3D Forest 0.42 released in 2017 is licensed under the terms of general public license (GNU GPL v3), is not platform specific, and is written in the C++ programming language. The source code, compiled version, user manual and a sample of testing data are available at the web page www.3dforest.eu. The compiled version is only available for the Windows 64 bit operating system. Hardware requirements are a 64-bit processor and at least 4 GB RAM memory. The application benefitted from using free libraries including: PCL [14], VTK [15], Boost [16], LibLAS [17] and Qt [18]. Only a brief description of the software algorithms follows, more details are available in the User Guide and the source code on the web site.

### Terrain extraction

Correct terrain extraction is of crucial importance, since most of the tree parameters are connected with a distance from the ground (DBH, tree height, etc.). Automated terrain extraction methods have been widely developed in ALS; in TLS processing, however, only a few studies have dealt with DTM extraction in forests in more detail [19, 20]. In 3D Forest, we implement two methods: i) segmentation of the lowest points on the *Z*-axis based on a search in an octree structure; and ii) voxelization of the input cloud and selection of the lowest voxels on the *Z*-axis as the terrain.

The first method using an octree search is more complex, recursively subdividing the 3D space of the point cloud into eight cubes (all axes are divided in half) until arriving at the specified resolution *R* (*R* = length of the cube edge). Using a too-coarse resolution leads to missing spots in the terrain, while a too-fine resolution leads to a very noisy terrain cloud. A two pass algorithm is incorporated for minimalizing noise points: In the first pass, a temporary rough sub-cloud containing all points of the lowest cubes of tenfold resolution (10*R*) is created. The purpose is to remove vegetation points from places where the true terrain points are missing (shadowed during scanning). Then the second pass takes place—a new octree search of resolution *R* is carried out within the first temporary sub-cloud and the result is saved into a new terrain point cloud, while the rest is saved as a vegetation point cloud. The octree search provides more detailed results, but with more noise included.

The second method calculates a centroid for points within every voxel of given resolution (defined by the user) and creates a new point cloud of voxel centroids. The centroids of the lowest voxels on the *Z*-axis are selected and saved as the terrain point cloud; the rest is classified as the vegetation point cloud.

The noise points in automatically segmented terrain (e.g. stumps, lying deadwood) can be removed using built-in filters or adjusted manually. Missing points in the terrain point cloud (e.g. in areas shielded by stems during scanning) can be filled in by inverse distance weighted interpolation, also incorporated in the application.

### Segmentation of trees

For the segmentation of forest vegetation into individual trees (Fig 1c), we developed an automatic approach based on the distance between points, minimal number of points forming clusters and the angle and distance between centroids of the clusters. In the first step of the segmentation, the entire vegetation is divided into horizontal slices with user-defined input distance *S* [cm] (*S* is a fundamental parameter of the segmentation and is also used in subsequent steps). Within these slices the clusters with a user-defined minimal number of points *N* and maximal distance *S* between the two nearest points are constructed. The next step is to reconstruct the bases of the trees. For each cluster with a centroid height lower than 1.3 m above terrain the 10 neighboring (nearest) clusters up to distance *2S* are found. We suppose those clusters come from the same tree base. All such clusters are merged into segments and tested if they are formed by at least five clusters and if the maximal dimension of the segment is at least 1 m to be identified as the tree. When all segments are tested and evaluated, we use a different approach to add more clusters to the tree. A cluster is added to the tree if its centroid lies within the distance *4S* to the nearest centroid of the tree and the angle between the vector of these two centroids and the Eigen vector of the 5 closest centroids of the stem is less than 10 degrees. For non-selected clusters we test the distance between cluster points and tree points and if the cluster fits the distance condition *S*, then the cluster is joined to the tree. In the final step, all non-selected points are tested to see if they can be joined to any tree according to gradually rising distance (maximally to *3S*). Automatically segmented trees can be visually checked and adjusted by manual segmentation if needed. Resulting individual tree clouds are used for estimations of tree parameters.

There are two issues that may appear to be the main limitations of our approach: i) based on the forest type, a high grass / herbaceous understory or dense thicket of small trees may be presented on the plot and after scanning in the point cloud. Those points may be interpreted as a tree, although they should actually be in the cloud of non-selected points; ii) tree parts (usually branches) may be connected to other tree. Those limitations are partly solved in the design of adding clusters to trees–trees are not treated at once, for each tree are added only the closest clusters based on a breadth-first search. Thus only the closest clusters meeting the segmentation conditions are added to the tree and then the other trees are treated before adding the next level of clusters to the same tree. Trees with the end of a branch that is lower than the beginning and trees extending into another tree are the most problematic–such branches are treated as a part of a different tree. For these cases the result of segmentation can be manually edited and redundant parts can be deleted from the wrong tree and added to the right one. The segmentation can also be supported by some method of proxy classification of clusters based on tree parameters (similar to [21]).

Our segmentation algorithm performs better in the leaf-off state, especially in dense stands, where the segmentation is generally trickier. In the leaf-on state there will be more obstacles and thus more shadows in the point cloud, which usually leads to worse tree segmentation. On the other hand, sparse forest stands can also be segmented well in the leaf-on state, as documented by [21].

We are aware of only two other automated methods of tree segmentation [21, 22]. Although their segmentation algorithms are different, the limitations of all methods are common: irregular point density due to shadows and overlapping trees and branches. The above-mentioned [21] uses a strategy consisting of three major parts: point cloud normalization, trunk detection and DBH estimation, and finally crown segmentation. Trunks are detected using density-based spatial clustering, and when all trunks are segmented, for each trunk the DBH and mean horizontal distance to its center is measured. Crown points are then segmented based on the weighted distance to the tree base and respective tree DBH. Raumonen et al. [22] use two main principles—tree topology and cover-sets. The segmentation of the point cloud into stems and branches is done using large surface patches of a fixed size. The stem bases and approximate stems are located heuristically, based on the assumption that stems are vertical.

### Tree parameters

Segmented trees are then ready for computing tree parameters. 3D Forest 0.42 can compute the following parameters: tree position, tree DBH, tree height / tree length, stem curve and tree planar projection (for crown parameters see section Crown parameters).

**Fig 2:**
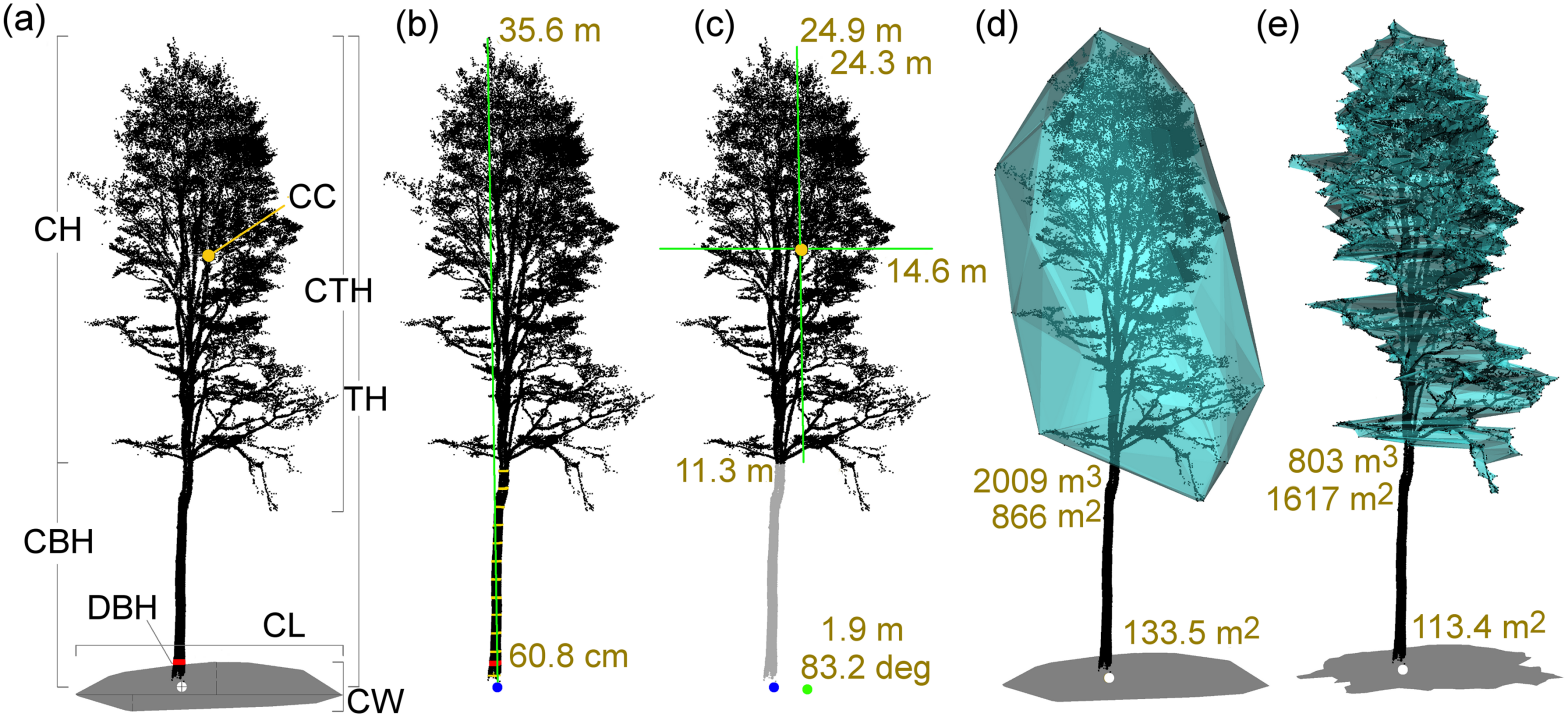
Extraction and visualization of tree parameters from a single tree cloud. (a) visualization of tree parameters: CBH–crown base height, CH–crown height, CTH–crown total height, CL–Crown length, CW–crown width, CC–crown centroid, DBH–diameter at breast height, TH–tree height, white sphere–tree position; (b) tree with computed basic parameters: position (blue sphere), DBH (60.8 cm), TH (green line; 35.6 m) and stem profile (yellow cylinders); (c) tree crown (black cloud) represented by CTH (24.9 m), CH (green line; 24.3 m), CL (green line; 14.6 m), CBH (11.3 m), crown centroid (orange sphere) and its planar projection (green sphere) with distance and azimuth from the tree position; (d) 3D convex hull of the crown with volume (2009 m^3^) and surface (866 m^2^) and orthogonal projection into plane with appropriate surface area (133.5 m^2^); (e) concave hull of the crown with volume (803 m^3^) and surface (1617 m^2^) and orthogonal projection into plane with its surface area (113.4 m^2^).

Tree position in censuses is usually understood as the position of the center of the tree base [23], and this convention was also adopted in 3D Forest (white sphere in Fig 2a). Two methods for extracting the tree position are implemented. The first method uses all points up to a user-specified height (default is 60 cm) above the lowest point of the tree and computes median coordinates of *X* and *Y*. The *Z* coordinate is defined as the median *Z* value of the *n* (default value is 5) closest points of the terrain to that *X*, *Y* position. The second method uses a similar approach as in [19]: we apply a Randomized Hough Transform (RHT) for circle detection [24] on tree points at 1.3 m and 0.65 m above the lowest point of the tree cloud. The tree position is defined as the intersection of the vector formed by centers of the two estimated circles with the DTM surface.

The two available methods for the computation of Tree DBH (red cylinder with size in cm in Fig 2a and 2b) are: i) RHT for circle detection with adjustable number of iterations (default is 200) of circle estimation [24]; and ii) Least Square Regression (LSR) with an algebraic estimation of the circle and geometric reduction of squared distances to the computed circle [25]. Both methods use a sub-set of the tree point cloud–a horizontal slice from 1.25 to 1.35 m above the calculated tree position—called the DBH cloud in the 3D Forest environment. For successful circle fitting at least 4 points in this slice are needed. Both methods have been tested for their sensitivity to input data and computational time in a manner to find the best use and setup for each method (see section Analysis of sensitivity for DBH computation). Manual editing (i.e. elimination of all points not representing the DBH) is available at this stage of the 3D Forest workflow.

The Tree Height (TH) is defined as the difference in *Z* coordinates between the highest point of the tree point cloud and the tree base position (vertical line and number above the tree in Fig 2a and 2b). The alternative method (Tree Length) computes the largest Euclidean distance between any two points of the tree point cloud. This method is thus suitable for calculation of the total length of leaning trees or even the length of lying deadwood.

For analysis of the Stem curve and its shape we use a similar approach as in [6]. The position of stem centers and stem diameters are calculated at different heights above the tree base position, starting at 0.65 m and followed by 1.3 m, 2 m and then every next meter above terrain (yellow cylinders in Fig 2b). The circles (defining the local stem center and diameter) are fitted by the RHT algorithm to horizontal 7 cm slices of the tree point cloud clipped at appropriate heights. The algorithm stops when the estimated diameter is two times greater than in both of the two previous circles, which indicates expansion of the tree cloud into the crown.

3D Forest can compute and visualize an area of Tree planar projection using a 2D convex/concave hull of the tree point cloud orthogonally projected onto the horizontal plane at the height of the tree base position. The convex hull (Fig 2d) is calculated using the Gift wrapping algorithm [26], and then the area of the resulting polygon is calculated. Since convex shapes do not fit well the actual shape of many irregular trees, we also implemented a concave planar projection (Fig 2e). The concave projection extends the convex hull algorithm using the Divide and conquer algorithm to split the sides of the polygon according to the given maximal polygon side length. The level of detail/generality as well as the area of the concave polygon can vary according to the maximal side length value defined by the user.

### Crown segmentation

In crown segmentation, tree clouds are separated into the stem and crown of a tree. This can be performed manually or using automatic extraction.

In the first step of the automatic extraction algorithm, the tree cloud is divided into 0.5m high horizontal sections and its widths (average *x* and *y* axis extension) are compared one by one from the lowest section. If three (or more) consecutive sections are wider than the previous one, the last thin section is used as a starting position for a detailed search. As the first step of the detailed search, the circles are fitted by LSR to two 10cm-high horizontal sections. From the centers of those circles the position of the fitted circle center of the next 10cm-high horizontal section is approximated. The subset of points for consecutive circle fitting is limited to the points within a radius two times greater than the last fitted circle. This should avoid using points representing overhanging branches for diameter computation. If the new (uppermost) section diameter is not 25% wider than the previous one, the algorithm continues by predicting the next stem center from the last two fitted circles and computing the next section diameter. The height of the last section diameter complying with the defined limit is considered as the crown base. All points of the tree cloud above this position are considered as the tree crown, together with points, that were excluded from fitting circles in the detailed search. LSR fitting is used here for its shorter execution time and especially for its sensitivity to outlying points that in effect detect places where branches are attached to the main stem (i.e. the crown base).

When the manual separation is used, all stem points below the crown are manually removed from the tree cloud. The *Z* coordinate of the highest point from the removed points is taken as the crown base height.

### Crown parameters

Similarly as for whole trees, specific parameters can also be computed for tree crowns. The parameters available in 3D Forest 0.42 are listed below with a brief description:

The Crown Base Height (CBH) is in relation to the tree position and might be defined as the height where the lowest branch is connected to the stem. This is computed as the difference between the tree base position *Z* coordinate and the *Z* coordinate of the crown base resulting from the crown extraction (Fig 2a and 2c).

Crown Height (CH) is the difference between the *Z* coordinate of the crown base and the *Z* coordinate of the highest point of the crown (Fig 2a and 2c).

Crown Total Height (CTH) represents the difference between the *Z* coordinates of the crown’s highest and lowest points (Fig 2a and 2c).

Crown Length (CL) is the longest distance between the two vertices of the convex hull of the crown planar projection (Fig 2a and 2c).

Crown Width (CW) is the sum of the two longest perpendicular distances from the crown length line to a convex hull vertex (Fig 2a).

Crown centroid (CC) is computed from border points, which are defined by 2D concave hulls of crown horizontal sections. The height of the horizontal sections and the maximal length of the concave hull edge are adjustable by the user. The position of the crown centroid is then computed as average coordinates from border points (orange point in Fig 2a and 2c); this avoids displacement of the crown centroid caused by a different cloud density when all crown points are used

Crown position deviation (CPD) is defined by the distance, direction (azimuth angle) and inclination. The distance and direction are measured between the tree base position and the orthogonal projection of the crown center position (green sphere in Fig 2c). Crown inclination is the inclination of the line connecting the tree base position and the position of the crown center from the vertical.

Crown volume and crown surface area may be estimated by its concave and/or convex 3D representation. The concave representation (Fig 2e) is based on horizontal sections (slices) of user-defined height and its concave hulls. Crown volume is then the sum of volumes of all horizontal sections (which are calculated as section 2D concave hull areas multiplied by the section height). Surface area is computed by a specific triangulation algorithm. In short, the triangulation is based on polygons created by the concave hull of each section (border points). The top and bottom of the crown is triangulated by creating triangles between the highest/lowest point of the crown and the highest/lowest polygon edges respectively. The rest is triangulated by strip triangulation of two consecutive polygons.

3D convex hull created by 3D Voronoi triangulation (Fig 2d) is the second option for calculating the crown volume and surface area. To reduce the calculation time only border points and all points from the two uppermost and lowermost horizontal sections are used implicitly. If needed, computation using all crown points is also available.

Calculation of crown volume by voxels of user-specified size is also available; crown volume is then the sum of voxels volumes. All voxels that contain at least one point are counted.

Last but not least, 3D Forest also allows users to calculate an intersecting mass of two neighboring crowns (Fig 1f). Intersection is computed as a Boolean AND in 3D space using objects created by 3D convex hull (only). The volume and center of mass of the intersection are computed. To provide additional information about the competition pressure in canopies, the direction from the crown centroid to the intersection center of mass is expressed by a horizontal azimuth and vertical angle; the distance in 3D space of these two points is also provided.

Because of the lack of reference values for actual crown metrics, the functionality of the algorithms for estimation of crown centroid, dimensions and convex and concave surface and volume was verified on various complex 3D geometrical shapes of known metrics (see Supporting information S1 Text and S1–S4 Figs). All verified parameters of defined 3D objects were estimated by 3D Forest with very high fidelity (S1 Table). We can therefore assume that the estimates of real crowns metrics are also reliable.

## Comparison with conventional measurements

To demonstrate the actual applicability of 3D Forest in real conditions, we compared outputs from 3D Forest with results from a standard census of the VPFDP. The VPFDP (10.3 ha) is a xerophilous forest on steep slopes and rocky outcrops characterized by highly variable canopy openness. The stand is dominated by sessile oak (*Quercus petraea* Matt.), with admixtures of European ash (*Fraxinus excelsior* L.), European hornbeam (*Carpinus betulus* L.), small-leaved lime (*Tilia cordata* Mill.), and 14 other tree species. The position and DBH of all trees with DBH ≥ 10cm were recorded in a census in 2013. Tree positions were measured by a Field-Map device (www.fieldmap.cz) using a regular grid (44 x 44 m) of reference points positioned by total station; DBHs were measured by a standard Haglöf caliper (recorded precision of 1cm). Tree heights were measured for 181 trees using a TruPulse laser rangefinder / digital inclinometer (recorded precision of 0.1 m).

At the same time, the whole plot was scanned in the leaf-off state using a Leica ScanStation C10 terrestrial laser scanner at a resolution of 2 mm in 10 m and using the regular multiple scanning position setup (44 x 44 m) as proposed by [27]. Scanned data were aligned, co-registered and exported into a txt file in the Cyclone Register software provided with the scanner. After importing of files and automated terrain/vegetation segmentation, 824 individual trees of DBH ≥ 10 cm on the 2.4 ha sub-plot were segmented in 3D Forest both manually and automatically from the vegetation point cloud. The DBH and height of segmented trees were automatically estimated by 3D Forest and compared to conventional field measurements.

### Automated tree segmentation

The first task was to evaluate the automatic tree segmentation algorithm depending on algorithm input parameters—input distance (S) of the cluster and minimal number of points (N) in the cluster. The outputs were compared in a confusion matrix with the manual tree segmentation used as a reference. The overall accuracy of the segmentation, mapping accuracy and omission and commission errors [28] for each tree were calculated on the bases of individual points of particular tree clouds. The best combination of input segmentation parameters was identified by a Kruskal-Wallis nonparametric ANOVA test (statistical significance level α = 0.05) and by pairwise comparisons using a post hoc Nemenyi test for pairwise comparisons with a Chi-squared approximation for independent samples. We tested the effect of combination of input parameters on tree segmentation accuracy expressed by mapping accuracy, commission error and omission error (see S2–S4 Tables).

**Fig 3:**
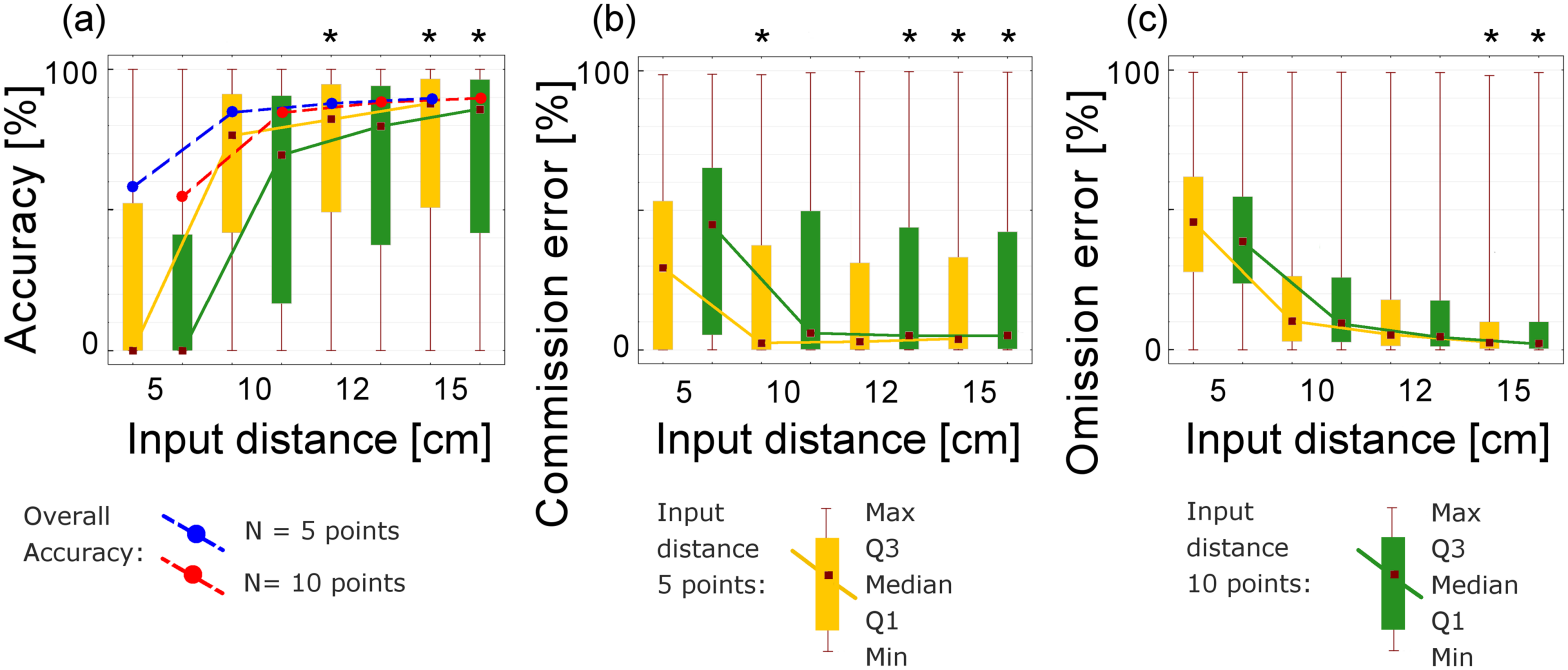
Accuracy of automated segmentation with different settings of *S* and *N*, as compared to manually segmented trees used as a reference. Four basic input distances (*S*) and two minimal numbers of points (*N*) were tested: (a) overall segmentation accuracy (blue dashed line for *N* of 5 points and red dashed line for *N* of 10 points in the cluster) and mapping accuracies of individual trees (boxplots and solid lines); (b) commission errors and (c) omission errors. In all charts square symbols connected by solid line represent medians, boxex upper and lower quartiles and whiskers represent upper and lower minimum and maximum; yellow color represents N of 5 points and green color N of 10 points in the cluster. Asterisks above boxplot mark settings that are statistically comparable to the best achieved result of the respective accuracy indicator according to the Nemenyi test.

Nonparametric ANOVA revealed that both factors—input distance (S) and number of points (N) had significant effects on the segmentation accuracy according to all three accuracy indicators–mapping accuracy, commission error and omission error (see S2–S4 Tables of the Supporting information). Fig 3 shows that overall and mapping accuracy rise with greater input distance. Overall accuracy gained 89.9% with the combination of 15 cm input distance (*S*) and minimum of 5 points (*N*) in the cluster; all setups with input distance greater than 5 cm achieved more than 85% overall accuracy. Since the overall accuracy gives us only general information about the whole segmentation (Fig 3a), we used the mapping accuracy of each tree as a more detailed descriptor of tree segmentation. The best mapping accuracy (median value 87.8%) was also achieved with an *S* of 15 cm and *N* of 5 points. Still, the post hoc test found no significant differences in mapping accuracy using *S* of 12 and15 cm and *N* of 5 points and using *S* of 15 cm and *N* of 10 points per cluster (settings marked by asterisks in Fig 3a). The median commission error decreased to the minimal value of 2.4% at *S* of 10 cm and *N* 5 points. However, *S* of 12 and 15cm at *N* of 10 points and *S* 15 cm at *N* 5 points per cluster were not significantly different from the best result (settings marked by asterisks in Fig 3b). The omission error (Fig 3c) had a similar trend as the commission error but smallest value (2.3%) was reached with *S* of 15 cm and *N* of 5 points. Settings *S* of 15 cm and *N* of 10 points was not significantly different from the best achieved result (see asterisks in Fig 3c). Taking all this into account, the input distance *S* of 15 cm and minimum number *N* of 5 and/or 10 points in a cluster provided the best segmentation results.

Our results were quite comparable with a similar study [21] even though the scanning setup and study area were different. Overall accuracy was comparable (90% vs 93%). The omission error (recall) and commission error (precision) were also similar, but 3D Forest achieved slightly better results in both errors (less than 2.5%) than the compared study (5%).

The optimal values of *S* and *N* can vary with the overall density of the TLS point cloud used—with more dense clouds the optimal segmentation distance can be smaller than 10 cm or clusters of more points might be preferable. Anyway, we have demonstrated that with appropriate settings the automatic segmentation algorithm may provide fairly acceptable results. Still, in closed canopy forests with an abundant understory and numerous stem and branch junctions of neighboring trees, a visual check and manual adjustment will be needed.

### Tree DBH and height

Since 3D Forest provides two methods of DBH estimation, we compared both methods with field measurements by paired t-tests; the pairs were arranged by joining the spatially nearest tree positions in both datasets. The tree height measurement was compared in the same way. The LSR method provided slightly higher values than conventional caliper measurements (mean difference 1.17 cm). The RHT method provided results quite comparable to the conventional field census (mean difference 0.3 cm; see Table 1). The tree heights derived by 3D Forest were comparable to conventional TruPulse field measurements, with a mean difference of 0.12 m (Table 1).

**Table 1:** Results of paired t-tests comparing automated methods of estimating DBH (Least Square Regression and Randomized Hough Transform) and tree height with conventional measurements using calipers and a digital inclinometer; computed for the significance level α = 0.05. Significant test is marked by asterisk in the last column.

Method	Units	Mean	Sdt. Dv.	N	Diff	Std. Dv. Diff	t	df	P	Confidence interval	
										-95%	+95%
**Manual DBH**	**cm**	32.30	9.06								
**LSR DBH**	**cm**	33.47	10.48	824	-1.17	5.34	-6.32	823	**0.00**	-1.54	-0.81*
**Manual DBH**	**cm**	32.30	9.06								
**RHT DBH**	**cm**	31.96	10.04	824	0.33	5.11	1.86	823	0.06	-0.02	0.68
**Manual height**	**m**	15.25	5.01								
**TLS height**	**m**	15.38	4.96	181	-0.12	1.62	-1.03	180	0.30	-0.36	0.11

Results of 3D Forest DBH estimations can be compared with another study [6] where DBH was evaluated. [6] achieved a slight overestimation of DBH using TLS (about 1 cm), similarly as by using the LSR method in 3D Forest. Conversely, the RHT method slightly underestimated the DBH (about 0.3 cm) but generally provided smaller differences from reference values than [6].

## Analysis of sensitivity for DBH computation

For the analysis of sensitivity we tried to find the optimal setup and limits of the two implemented methods of DBH computation. Five factors likely affecting correct DBH estimates were selected for testing: the stem diameter (D), a missing part of the DBH ring (M), the percentage of noise points (N) and the number of points (P) creating the DBH ring. For the RHT computation the number of iterations (I) was also tested, and for both methods the time required for computation evaluated.

The testing was performed on artificial dataset designed as a pooled sample of simulated DBH rings composed of points representing mixture of different levels of all tested factors. The factor of stem diameter was tested in the range from 1 to 500 cm (25 levels); a missing part of the DBH ring was defined as an angular percentage of the ring perimeter not covered by TLS points (tested at 10 levels in 0–90% range); the percentage of noise points was defined as the proportion of outliers (i.e. points not representing the circular stem perimeter) from the total number of points in the range 0–90% (10 levels); and the factor number of points was defined as the number of points in the DBH cloud used for the circle fitting (from 3 to 500 in 21 levels). The number of iterations was tested at 9 levels (10, 20, 30, 50, 100, 200, 300, 500 and 1000). Full factorial design was used to simulate a total of 48 300 DBH rings; the diameter of each ring (or its part) was estimated by both methods implemented in 3D Forest and compared to the expected value. A correct DBH estimation was rigorously defined as ± 0.1 cm difference from the expected value. The effect of factors on the probability of a correct DBH estimate was modeled by logistic regression (Table 2). The goodness of fit was evaluated by analysis of deviance (see S5 Table of the Supporting information).

**Table 2:** Results of logistic regression fit for all factors of DBH computation by both methods. Significant tests (at significance level α = 0.05) are marked by asterisks in the last column.

Method	Factors:	Estimate	Std. Error	z value	Pr(>|z|)
**LSR**	(Intercept)	21.801	7.26E+02	0.030	0.976
	Percentage of Noise (N)	-4.321	6.11E+01	-0.071	0.944
	Diameter (D)	0.000	2.26E+00	0.000	1.000
	Number of Points (P)	0.000	2.20E+00	0.000	1.000
	Missing part (M)	0.000	1.05E+01	0.000	1.000
**RHT**	(Intercept)	3.658	1.39E-02	262.570	2.2E-16*
	Percentage of Noise (N)	-0.069	2.02E-04	-340.730	2.2E-16*
	Diameter (D)	0.001	3.20E-05	42.390	2.2E-16*
	Number of Points (P)	-0.001	3.07E-05	-39.630	2.2E-16*
	Missing part (M)	-0.014	1.49E-04	-95.910	2.2E-16*
	Number of Iterations (I)	0.002	1.54E-05	158.57	2.2E-16*

The effect of factors on the probability of correct DBH estimate may be expressed for both methods by following formulas of the logistic function of probability resulting from the logistic regression:

$$\text{π}\left(\text{LSR}\right)=\frac{{\text{e}}^{\left(21.8-4.321*\text{N}\right)}}{1+{\text{e}}^{\left(21.8-4.321*\text{N}\right)}}$$

$$\text{π}\left(\text{RHT}\right)=\frac{{\text{e}}^{\left(3.658-0.069*\text{N}+0.001*\text{D}-0.001*\text{P}-0.014*\text{M}+0.002*\text{I}\right)}}{1+{\text{e}}^{\left(3.658-0.069*\text{N}+0.001*\text{D}-0.001*\text{P}-0.014*\text{M}+0.002*\text{I}\right)}}$$

where the coefficients are as follows: D—diameter of estimated DBH ring, P—number of points forming the ring, M—missing part of the ring (as percentage), N—percentage of noise points in the ring and I—number of iterations.

**Fig 4:**
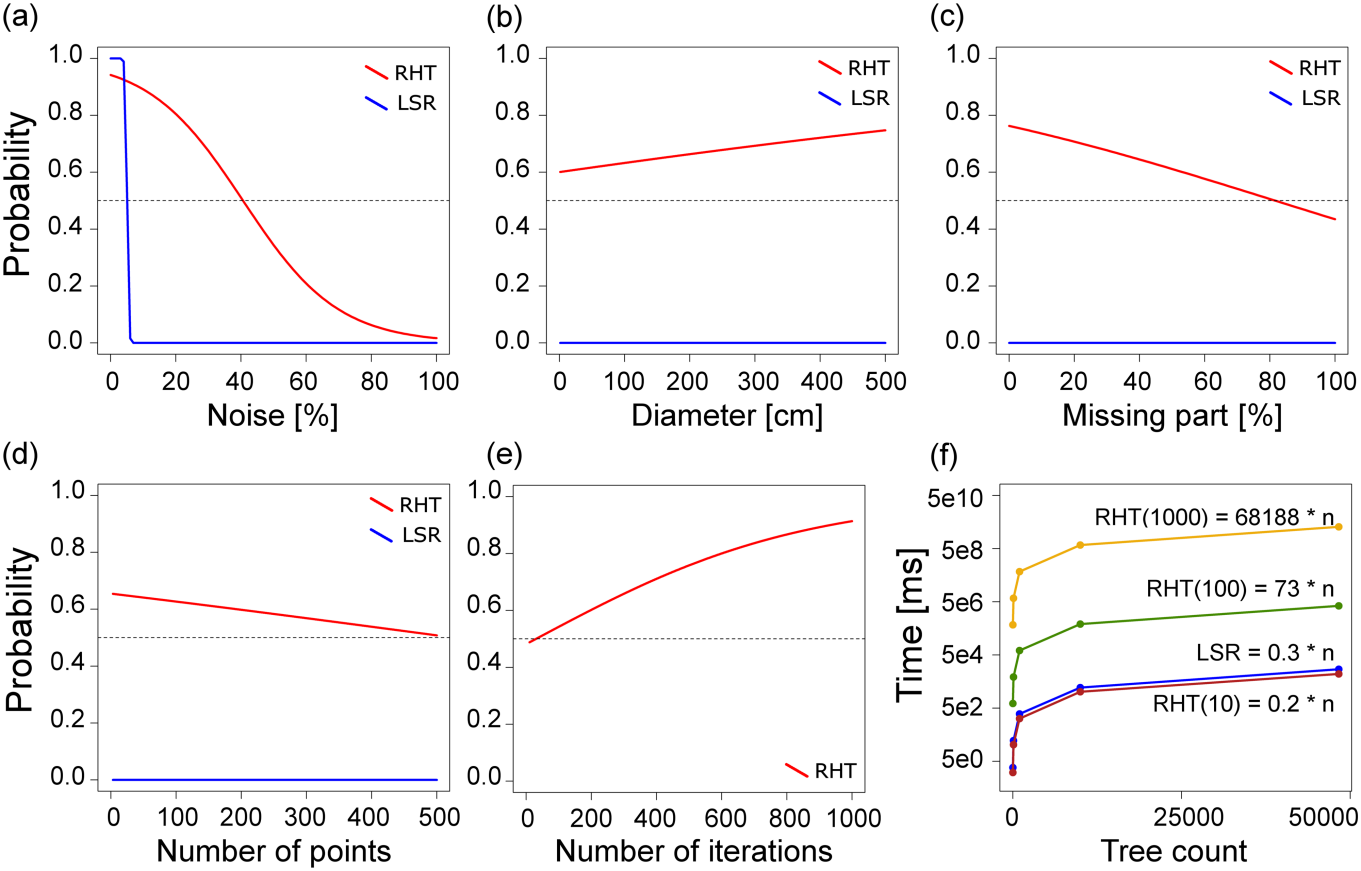
The effect of different factors on DBH computation analyses for both methods by logistic regression: RHT in red, LSR in blue. Factors: (a) percentage of noise points in the DBH ring (N); (b) diameter of the ring (D); (c) missing part of the ring (M); (d) number of points forming the ring (P); (e) number of iterations (I); (f) time required for DBH computation in relation to the number of trees and iterations (Y axis in logarithmic scale).

For the LSR method, only the noise factor proved to be crucial even though the test was not statistically significant (Table 2). This method is extremely sensitive to the presence of noise. Without noise, the LSR estimate was 100% correct, but already at 10% of noise points the probability of DBH estimation decreased to minimum (Fig 4a) and resulted in a total failure of the correct DBH estimation (Fig 4b, 4c and 4d). None of the other factors had an impact on the probability of a correct DBH estimate, so if only circumferential points of the stem are presented in the DBH cloud, tree DBH can be estimated correctly even from a very small part of the stem perimeter, irrespective of the stem diameter and number of points in the DBH cloud (at least 4 points are needed) and in a relatively short time (Fig 4f).

On the contrary, the RHT method was significantly affected by all tested factors (Table 2); the probability of a correct DBH estimate was the most affected by the presence of noise, the number of iterations and a missing part of the DBH ring respectively (see Table 2 and S5 Table). Yet, the method proved to be more robust and resistant to the presence of noise—the probability of a correct DBH estimation decreased relatively slowly with the initial increase of noise points (Fig 4a). Without noise the probability of a correct DBH estimate was above 90%, and still reached a level of 80% in the presence of 20% noise points. The probability of a correct DBH estimation gently increased with increasing diameter (Fig 4b); still, even the smallest diameters had the probability of a correct estimate above 60%. The probability of a correct DBH estimate decreased significantly with a missing part of stem perimeter (Fig 4c) and gradually also with the growing number of points forming the DBH cloud (Fig 4d). Conversely, the number of iterations significantly increased the probability of a correct DBH estimate, though above 500 iterations the increase in probability was relatively slower (Fig 4e), while the computation time increased by nearly cubic exponent with the number of iterations (Fig 4f); higher numbers of iterations are thus ineffective.

In our testing (Fig 4f), the time required of DBH computation (*y* [ms]) may be summarized for the LSR method as:

y(LSR)=0.3*n

and for the RHT method as:

$$y\left(RHT\right)=0.0002*n*i^{2.766}$$

where *n* is number of trees and *i* is the number of iterations. Still, the time was measured only on one machine and the actual computational time experienced may change with the various setups of different computers.

Due to the overall slight overestimation of the LSR method (Table 1) and the rigorous difference from the expected DBH value (± 0.1 cm) used in the definition of the correct DBH estimate for the sensitivity analysis, the LSR provided a generally significantly lower probability of successful DBH recognition there. The limiting factor is the presence of noise points in in the DBH cloud usually formed by branches of leaves around the tree stem at breast height. Presence of such noise then usually causes DBH overestimation. On the other hand, if the target trees do not have low branches (i.e. no noise in the DBH point cloud), LSR method can be more time efficient than using RHT (Fig 4f).

## Conclusions

The use of LiDAR technology undoubtedly has great potential in forest ecosystem research. While for processing ALS data several software packages may be used (e.g. FUSION, TerraScan, LiForest), only few free software applications specialized for tree descriptions are as yet available for TLS data. 3D Forest contributes to filling this gap by focusing on forest stand descriptions by means of individual trees and their mutual spatial arrangement in 3D space. It allows users to take advantage of TLS data for detailed spatially-oriented silviculture and forest ecology studies in a user-friendly environment. Currently it can produce standard census data such as tree positions, DBHs and heights, and also provides more complex tree parameters such as stem curve, convex/concave planar projection, crown dimensions, crown volume, surface, crown centroid and others. The methods implemented are comparable with other findings in the literature on the automatic segmentation of trees [21] and estimation of tree parameters [6] or crown parameters [29].

In addition, 3D Forest allows computation of the intersecting mass of two neighboring crowns, which affords superior identification and quantification of the aboveground competition of trees e.g. [9, 10] or testing the canopy-related predictions of the metabolic scaling theory of forests [29, 30] with authentic data on tree crowns. On the other hand, 3D Forest is still rather limited in pinpoint biomass estimates and detailed modeling of a single tree on the branch and leaf level [3, 22], as provided e.g. by SimpleTree [10].

3D Forest was primarily designed for the purposes of forest ecology and for application in natural forests with complex stand structure. Nevertheless, from the production forestry standpoint the performance of 3D Forest might also be satisfactory, because precise estimates of timber volume and quality in mature stands are usually required before logging. These stands are usually of simple structure and lack low branches (depending on the forest type and the silviculture system used). Therefore, successful tree segmentation and parametrization (e.g. recognition of DBH, tree height, canopy base height and stem curve) may be anticipated in such forests. In a selection silviculture system, the stand structure may be more complex, but again, the focus of forestry is on target (mature) trees, which are easier to segment and process.

The future development of 3D Forest is aimed at realistic modeling of the potential direct solar irradiance of individual tree crowns based on real tree shapes, positions and the sun path at the respective latitude, year season and daytime. This will enable studies of competition for light in a new individualistic manner (e.g. which tree crown overshadows which, when and how much).

The 3D Forest software including the source code is freely available at www.3dforest.eu. Researchers and developers are openly invited to join our effort in the further development of the software. 3D Forest users can also leave comments and suggestions at the forum or via a ticket system on the web page.

## Supporting information

S1 Dataset Dataset for all analysis presented in paper. Zipped files with data for analysis. File Automatic_segmentation-data.xlsx contains results of automatic segmentation. File DBH_height-data.xlsx contains manual data and corresponding computed data from 3D Forest. File Sensitivity-results-LSR.xlsx contains result of sensitivity analysis with all factors for LSR method. Sensitivity-results-RHT.xlsx contains data for RHT method and file Sensitivity-results-time.xlsx contains time measurement of each method.
(ZIP)

S1 Fig Simple convex geometrical objects with computed convex hull. Point clouds (black dots) arranged in simple convex geometrical 3D objects of known metrics represented by 3D convex hulls produced by 3D Forest (blue surface).
(PNG)

S2 Fig Simple convex geometrical objects with computed concave hull. Point clouds (black dots) arranged in simple convex geometrical 3D objects of known metrics represented by concave triangulation by 0.1m horizontal sections provided by 3D Forest (blue surface).
(PNG)

S3 Fig Complex concave geometrical objects with computed convex hull. Point clouds (black dots) arranged in complex concave geometrical 3D objects of known metrics represented by 3D convex hull made in 3D Forest (blue surface).
(PNG)

S4 Fig Complex concave geometrical objects with computed concave hull. Point clouds (black dots) arranged in complex concave geometrical 3D objects of known metrics represented by concave triangulation by 0.1m horizontal sections provided by 3D Forest (blue surface).
(PNG)

S1 Table Various parameters of 3D shapes as calculated by 3D Forest and compared to reference values. Basic parameters (height, length and width), planar projection, surface and volume of 3D geometrical shapes computed by 3D Forest and compared to reference values.
(XLSX)

S2 Table The effect of factors S and N on the mapping accuracy of tree segmentation. Bold numbers denote statistically significant results. Asterisks denote the group of best segmentation settings (factor levels) according to Nemenyi post hoc test.
(XLSX)

S3 Table The effect of factors S and N on the commission error of tree segmentation. Bold numbers denote statistically significant results. Asterisks denote the group of best segmentation settings (factor levels) according to Nemenyi post hoc test.
(XLSX)

S4 Table The effect of factors S and N on the omission error of the tree segmentation. Bold numbers denote statistically significant results. Asterisks denote the group of best segmentation settings (factor levels) according to Nemenyi post hoc test.
(XLSX)

S5 Table Analysis of sensitivity of DBH computation using logistic regression- goodness of fit. The goodness of fit was evaluated using analysis of deviance table where deviance column gives difference between models as variables (i.e. factors) are added to the model in turn.
(XLSX)

S1 Text Verification of tree crown metrics provided by 3D Forest. (DOCX)
