# Supplementary figures and images for: 3D Forest: An application for descriptions of three-dimensional forest structures using terrestrial LiDAR
Source: PLoS One. 2017 May 4;12(5):e0176871. doi: 10.1371/journal.pone.0176871 (PMC5417521; doi:10.1371/journal.pone.0176871)

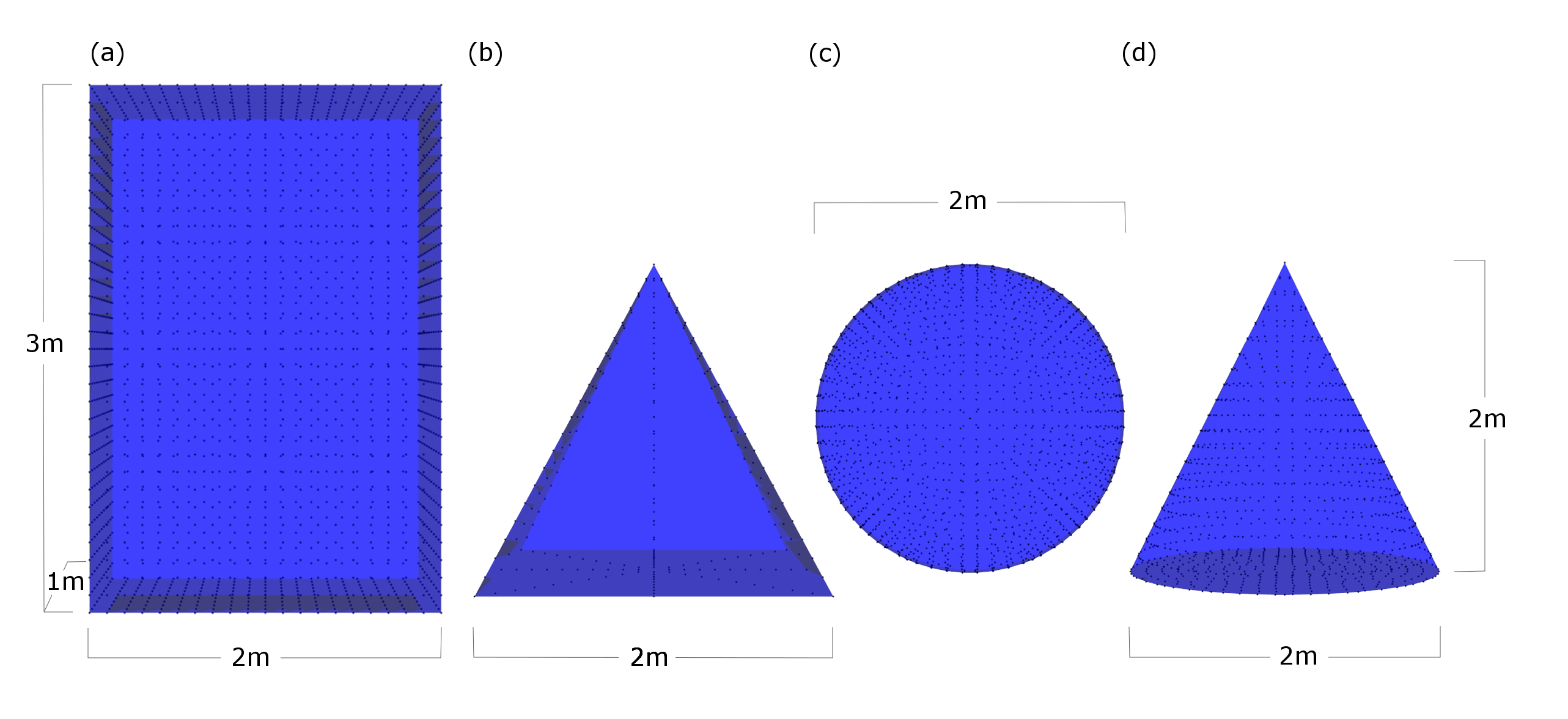

Supplement: S1 Fig — Point clouds (black dots) arranged in simple convex geometrical 3D objects of known metrics represented by 3D convex hulls produced by 3D Forest (blue surface). (PNG) [file pone.0176871.s002.png]

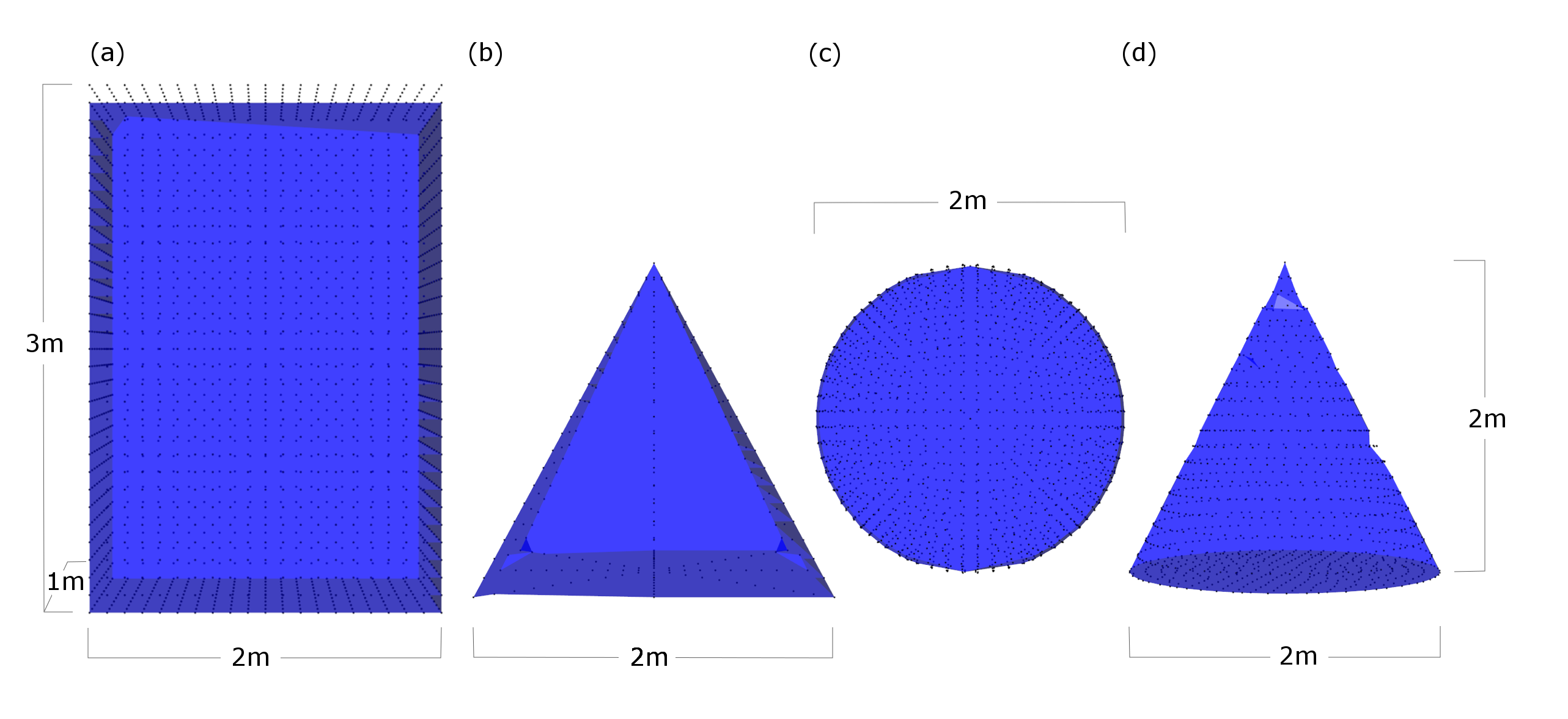

Supplement: S2 Fig — Point clouds (black dots) arranged in simple convex geometrical 3D objects of known metrics represented by concave triangulation by 0.1m horizontal sections provided by 3D Forest (blue surface). (PNG) [file pone.0176871.s003.png]

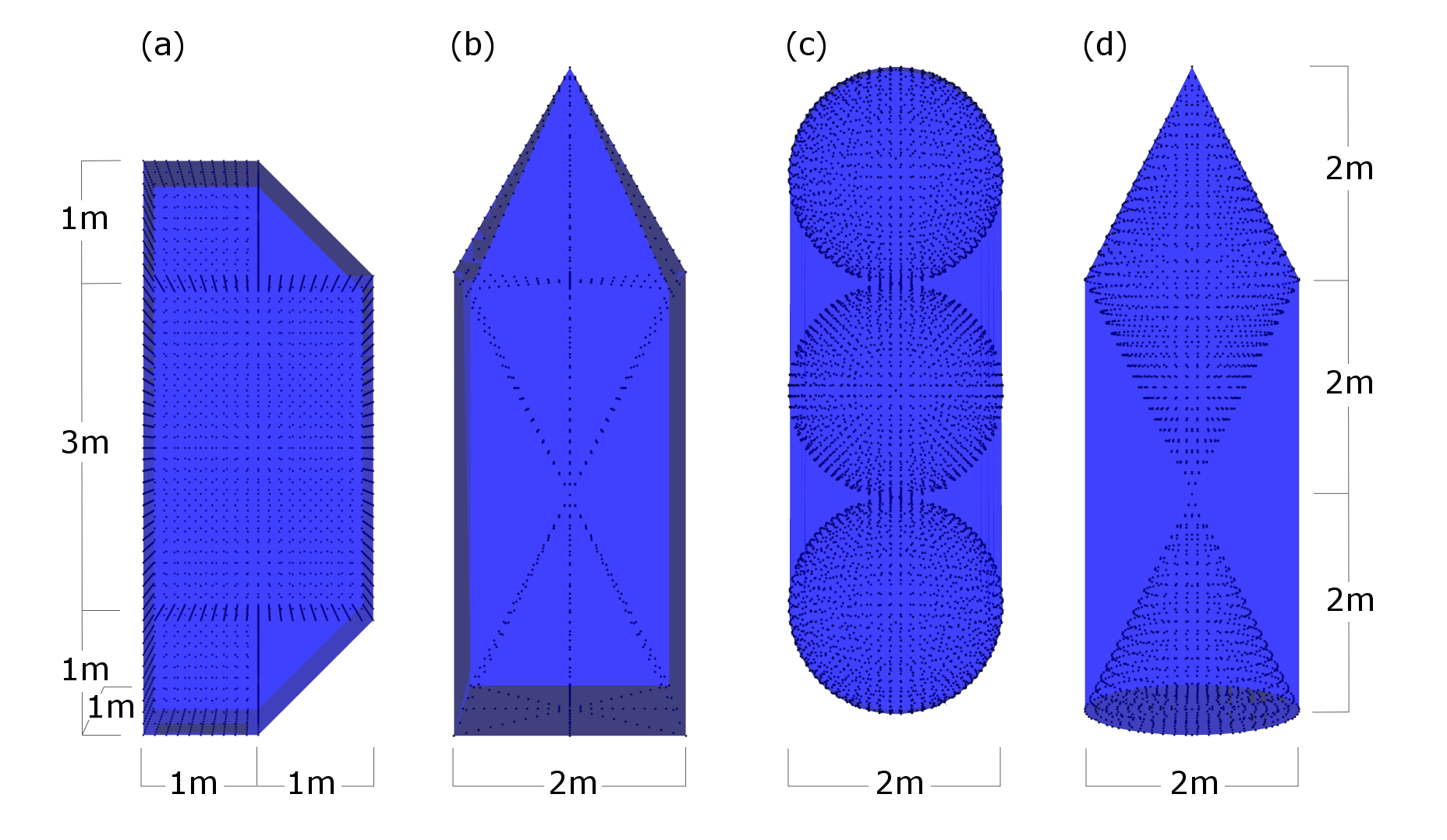

Supplement: S3 Fig — Point clouds (black dots) arranged in complex concave geometrical 3D objects of known metrics represented by 3D convex hull made in 3D Forest (blue surface). (PNG) [file pone.0176871.s004.png]

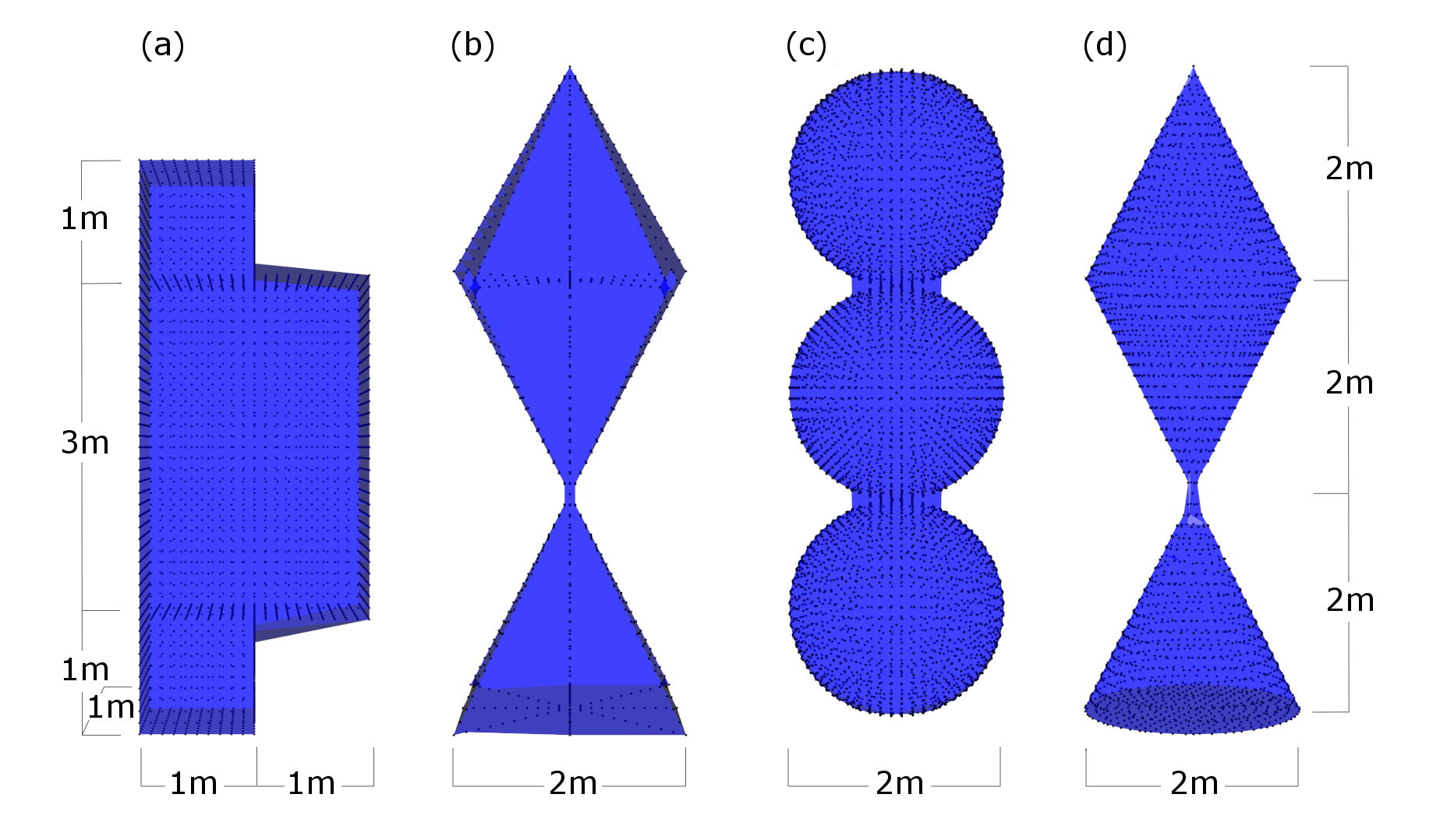

Supplement: S4 Fig — Point clouds (black dots) arranged in complex concave geometrical 3D objects of known metrics represented by concave triangulation by 0.1m horizontal sections provided by 3D Forest (blue surface). (PNG) [file pone.0176871.s005.png]
