# Supplementary material for: 3D Forest: An application for descriptions of three-dimensional forest structures using terrestrial LiDAR
Source: PLoS One. 2017 May 4;12(5):e0176871. doi: 10.1371/journal.pone.0176871 (PMC5417521; doi:10.1371/journal.pone.0176871)
Supplement: S1 Text — (DOCX) [file pone.0176871.s011.docx]

**Verification of tree crown metrics provided by 3D Forest**

Calculation of crown metrics from point clouds were verified on four simple convex 3D geometrical objects (block, pyramid, sphere and cone - S1 and S2 Figs.) and four composed concave 3D geometrical objects (3 blocks, 3 pyramids, 3 spheres and 3 cones stacked vertically – S3 and S4 Figs.). All defined objects were of known dimensions. In each group of objects (convex / concave) two were of angular shape (a, b) and other two of round shape (c, d). The following crown metrics were evaluated: (total) height, length and width, projected area, surface and volume.

As demonstrated in S1 Table, the basic dimensions of all 3D objects were calculated with absolute accuracy. This was clearly expected, because the object dimensions are just distances between points in Euclidean space. The planar projected area of angular 3D objects was in 3D Forest also calculated with absolute accuracy (S1 Table). There was a small underestimation (in the order of tenths of a percent) for round objects only, because the  convex/concave hull used in 3D Forest for projected area calculations is based on straight lines, not curves. There was no difference between simple and complex shapes, as their planar projections were identical.

Similarly, convex surfaces of angular objects (both simple and/or composed) were calculated in 3D Forest with absolute accuracy (S1 Table). Convex surfaces of round objects slightly deviated from reference values (on the order of tenths of a percent), because in 3D Forest the surface is based on triangulation, which is always imperfect for round objects always imperfect. For concave surfaces the differences were slightly higher and appeared in all object types, since the definition of concave objects in 3D Forest is based on strip triangulation between vertical sections. The height of the section (as well as the maximal length of its concave hull edge) naturally affects the detail of object definition. In our test we used 0.1m high horizontal sections and a maximal length of the concave hull edge of 1 m.

A quite analogical pattern might be observed for object volumes. Convex volumes of angular objects (both simple and/or composed) were calculated in 3D Forest with absolute accuracy (S1 Table). Convex volumes of round objects slightly deviated from reference values, because in 3D Forest the objects are defined by triangulation, which is always imperfect for round objects. For concave volumes the differences were slightly higher and appeared in all object types. This is because in 3D Forest the concave volume is calculated by horizontal sections (the concave projected area of the section multiplied by the section height) and the height of the section (as well as the maximal length of its concave hull edge) affects the details of volume calculation. In this test we used 0.1m high horizontal sections and a maximal length of concave hull edge of 1 m.

In general, the volume and surface of complex concave 3D objects such as real tree crowns may vary substantially just due to the level of detail of their definition. In our view, the accuracy of surface and volume estimates provided by 3D Forest is thus entirely sufficient.
